# Immunomodulatory effects of mesenchymal stem cell-conditioned media on lipopolysaccharide of *Vibrio cholerae* as a vaccine candidate

**DOI:** 10.1186/s13287-021-02622-0

**Published:** 2021-11-03

**Authors:** Mahboube Bahroudi, Bita Bakhshi, Sara Soudi, Shahin Najar-peerayeh

**Affiliations:** 1 Department of Bacteriology, Faculty of Medical Sciences, Tarbiat Modares University, Jalal-Ale-Ahmad Ave., 14117-13116 Tehran, Iran; 2 Department of Immunology, Faculty of Medical Sciences, Tarbiat Modares University, Jalal-Ale-Ahmad Ave., 14117-13116 Tehran, Iran

**Keywords:** LPS, *Vibrio cholerae*, Mesenchymal stem cells, Cytokine, Antibody

## Abstract

**Background:**

*Vibrio cholerae* is the causative agent of cholera, which is commonly associated with high morbidity and mortality, and presents a major challenge to healthcare systems throughout the world. Lipopolysaccharide (LPS) is required for full protection against *V. cholerae* but can induce inflammation and septic shock. Mesenchymal stem cells (MSCs) are currently used to treat infectious and inflammatory diseases. Therefore, this study aimed to evaluate the immune-modulating effects of the LPS‐MSC‐conditioned medium (CM) on *V. cholerae* LPS immunization in a murine model.

**Methods:**

After preconditioning MSCs with LPS, mice were immunized intraperitoneally on days 0 and 14 with the following combinations: LPS + LPS-MSC-CM; detoxified LPS (DLPS) + MSC-CM; LPS + MSC sup; LPS; LPS-MSC-CM; MSC supernatant (MSC sup); and PBS. The mouse serum and saliva samples were collected to evaluate antibody (serum IgG and saliva IgA) and cytokine responses (TNF-α, IL-10, IL-6, TGF-β, IL-4, IL-5, and B-cell activating factor (BAFF)).

**Results:**

The LPS + LPS-MSC-CM significantly increased total IgG and IgA compared to other combinations (*P* < 0.001). TNF-α levels, in contrast to IL-10 and TGF-β, were reduced significantly in mice receiving the LPS + LPS-MSC-CM compared to mice receiving only LPS. IL-4, IL-5, and BAFF levels significantly increased in mice receiving increased doses of LPS + LPS-MSC-CM compared to those who received only LPS. The highest vibriocidal antibody titer (1:64) was observed in LPS + LPS-MSC-CM-immunized mice and resulted in a significant improvement in survival in infant mice infected by *V. cholerae* O1.

**Conclusions:**

The LPS-MSC-CM modulates the immune response to *V. cholerae* LPS by regulating inflammatory and anti-inflammatory responses and inducing vibriocidal antibodies, which protect neonate mice against *V. cholerae* infection.

**Supplementary Information:**

The online version contains supplementary material available at 10.1186/s13287-021-02622-0.

## Introduction

Cholera, which is caused by *Vibrio cholerae*, is endemic in many regions of the world [1, 2]. According to the World Health Organization (WHO), between 5 and 7 million people are infected with the disease annually, leading to more than 100,000 to 130,000 deaths worldwide (www.who.int/mediacentre/factsheets/fs107/en/index.html). Moreover, the emergence of multi-drug resistance (MDR) in *V. cholerae* strains has become a severe concern in developing countries [3, 4]. The high mortality rate from cholera and lack of effective antimicrobial agents [4–6] highlight an undoubted need for new, nonantibiotic approaches effective against drug-resistant strains. Currently, the WHO has prequalified three whole-cell killed oral cholera vaccines: Dukoral®, which can be given to all individuals older than 2 years, and Shanchol™ and Euvichol®, which can be given to all individuals older than 1 year [5, 6]. However, these vaccines do not provide complete long-term protection and require two doses two weeks apart with a booster every 2 years. [5, 6]. Moreover, children younger than 2 years exhibit a less effective and less durable immune response to these vaccines. None of these vaccines have been approved for use in children younger than 1 year [2, 7].

*Vibrio cholerae* strains are distinguished serologically by the presence of the O-antigen, a component of the surface lipopolysaccharide (LPS) of the bacterial cell [8]. Most *V. cholerae* strains causing epidemic cholera in many countries typically belong to the serogroup O1 or O139 [9]. The *V. cholerae* LPS is an immunogenic antigen that induces significant increases in serum IgG, IgM, and IgA responses, as well as antibody-secreting cell responses in the human host [2, 10–12]. *Vibrio cholerae* O1 LPS of the IgA isotype is considered important in protecting the individual from the disease [13, 14]. In response to *V. cholerae* LPS, the host mucosal immune cells induce inflammatory cytokines (TNF-α and IL-6) and inhibit anti-inflammatory cytokines (IL-10 and TGF-β), which increase *V. cholerae* intestinal pathology and invasion [2, 15, 16]. An effective therapeutic approach is one that can modulate the production of inflammatory cytokines following *V. cholerae* infection. Such an approach will help treat and manage an infection caused by MDR *V. cholerae*, which is major challenge to healthcare systems worldwide.

Human mesenchymal stem cells (MSCs) are multipotent cells capable of proliferation and self-renewal and also differentiate into several cell types, such as osteoblasts, chondrocytes, and adipocytes [17, 18]. Several studies have highlighted potential of MSCs to modulate innate immune cells by inducing a wide range of cytokines and immunomodulatory mediators [19–21]. MSCs also secrete diverse bioactive compounds having anti-inflammatory, antimicrobial, chemotactic, and antiapoptotic properties [19–21]. Moreover, MSCs promote macrophage and endothelial-cell recruitment to the injured or infected site [20, 22, 23], regenerate damaged tissues, and improve immune responses against bacteria by modulating the secretion of inflammatory and anti-inflammatory cytokines [24–26]. In the present study, we sought to determine the immune-modulatory effects of the LPS-MSC-conditioned medium (CM) on *V. cholerae* LPS in a murine model. The efficacy of the vaccine was examined by determining pro- and anti-inflammatory cytokines and vibriocidal antibodies in infected mice.

## Material and methods

### Bacterial strain and culture

LPS from *V. cholerae* O1 ATCC 14,035 was used in the mouse challenge experiment. *Vibrio cholerae* O1 was cultured in the brain heart infusion (BHI) broth and Luria–Bertani (LB) broth (all from Merck, Germany). The bacterial strain in the LB broth was cultured for 24 h to an optical density (OD) of 1:0, equivalent to 10^8^ colony-forming units (CFU)/mL.

### Mice

Female BALB/c mice, aged 6–8 weeks and 6 days, were purchased from the Royan Institute (Tehran, Iran). All animal experiments were conducted in accordance with the protocols approved by the Animal Ethics Committee of Tarbiat Modares University (approval number: IR.MODARES.REC.)

### Extraction of *V. cholerae* LPS

LPS from *V. cholerae* O1 was extracted using an LPS extraction kit (iNtRON Biotechnology, Seongnam, Korea), according to the manufacturer’s instructions. LPS from *V. cholerae* O1 was extracted using an LPS extraction kit (iNtRON Biotechnology, Seongnam, Korea), according to the manufacturer’s instructions. Briefly, bacterial cultures were centrifuged and re-suspended in lysis buffer and vortexed vigorously to dissolve cell clumps. Then, the suspension was mixed with chloroform and centrifuged at 13,000 rpm for 10 min at 4 °C. Then, the aqueous layer was mixed with purification buffer and was centrifuged. The pellet was washed with 70% ethanol and centrifuged at 13,000 rpm for 3 min at 4 °C. The pellet was dried at room temperature and dissolved in 10 mM Tris–HCl (pH 8.0) by boiling for 2 min. The extracted LPS was fractionated using SDS‐PAGE. The SDS-PAGE gel was then submitted to silver staining (Additional file 1: Fig. S1). The silver-stained SDS-PAGE of the LPS extract demonstrated two bands of molecular weights ~ 35 and ~ 15 kDa, corresponding to antigen-O and lipid A core, respectively (Additional file 1: Fig. S1). For the in vivo experiment, the extracted LPS was detoxified (DLPS) by alkaline treatment, as described previously [27].

### Preparation of MSCs

MSCs obtained from Bon Yakhteh (Tehran-Iran) were cultured in the DMEM high-glucose medium supplemented with 10% FBS, 2 mM L-glutamine, and penicillin–streptomycin (1x) (all from Gibco, USA) at 37 °C in 5% CO_2_. Expression of MSC-specific surface antigens CD44, CD73, CD90, CD105, and CD44, and the absence of CD31, CD34, and CD45, was confirmed by flow cytometry using specific antibodies, as previously described [28]. To determine the immune profile of MSCs according to the International Society for Cellular Therapy (ISCT) standards, 1 × 10^5^ cells/mL were stained with PE (phycoerythrin)-conjugated antibodies against CD44, CD73, CD90, CD105, CD31, CD34, and CD45 (all from ebioscience, Germany) and were analyzed on FACS flow cytometry using Cell Quest Software (Becton Dickinson, UK) [11]. MSCs were positive for CD44, CD73, CD90, and CD105 but were negative for CD31, CD34, and CD45. The multipotency of MSCs was confirmed by osteogenic, chondrogenic, and adipogenic differentiation [11]. Osteogenic differentiation of MSCs was induced in a Dulbecco’s modified Eagle’s medium (DMEM) supplemented with 10% FBS, 10 mM β-glycerophosphate (Sigma, USA), 100 nM dexamethasone (Sigma, USA), and 100 mM L-ascorbic acid. Medium was changed every 3 days. On day 7, the cells were harvested for alkaline phosphatase staining[ 29]. For adipogenic differentiation, MSCs were incubated in a DMEM supplemented with 1 µM dexamethasone, 0.5 mM isobutylmethylxanthine, 10 nM insulin and 100 µM indomethacin at 37 °C for 12 days, and the media were changed every 3 days. Then, the cells were harvested for oil red O staining [30, 31].

### Optimization of preconditioning procedure of MSCs with LPS

MSC viability after preconditioning with different concentrations of LPS was evaluated using the 3-(4,5-dimethylthiazol-2-yl)-2,5-diphenyltetrazolium bromide (MTT) assay, as was previously described [32, 33]. To determine the cytoprotective dose of LPS, 1 × 10^4^ MSCs/mL were treated with 0.5, 1, and 5 µg/mL LPS for 12, 24, and 48 h. The cells were incubated in the MTT solution (Sigma-Aldrich; USA), which was then replaced with dimethyl sulfoxide (DMSO, Sigma-Aldrich). The absorbance was measured at 570 nm. The percentage of cytotoxicity activity was calculated using the following formula: cytotoxicity activity (%) = absorbance of the experimental well/absorbance of the negative control well × 100.

To determine the optimal time for preconditioning MSCs with LPS, MSCs at 5 × 10^6^ cells/well were treated with 5 µg/mL as a cytoprotective dose of LPS for 24, 48, and 72 h. Then, the levels of pro- and anti-inflammatory cytokines, including IL-6, TNF-α, IL-10, and TGF-β, in the LPS-MSC-CM were measured.

### Mouse immunizations

Mice were randomly distributed into seven experimental groups and immunized intraperitoneally on days 0 and 14. Vaccine preparations per mouse included the following: 1] LPS + LPS-MSC-CM; 2] LPS + MSC sup; 3] DLPS + MSC-CM; 4) LPS; 5] LPS-MSC-CM. In the experimental groups, the concentration of LPS was 5 µg and volume of injected LPS-MSC-CM or MSC sup was 100 µL. Two control groups included: 6) the MSC sup mice, which were injected with the supernatant of nonconditioned MSCs; and 7) the PBS mice, which were mock-immunized with sterile PBS (100 µL). The mouse serum and saliva samples were collected on days 1 and 14 to evaluate antibody and cytokine responses.

### Measurement of antibody responses

Antigen-specific antibody titers against *V. cholerae* LPS were assessed by ELISA, as described previously [34, 35]. Briefly, each ELISA plate well (Nunc, USA) was coated with *V. cholerae* LPS at 5 µg/mL in PBS, incubated overnight at 4 °C, washed with 0.5% Tween-PBS (T-PBS), and blocked with PBS + 3% bovine serum albumin (Sigma-Aldrich). Mouse serum or salvia was incubated overnight on the plates at 4 °C and washed 3 times with T-PBS, followed by addition of 100 µL HRP-conjugated anti-mouse IgA or IgG antibody (Sigma-Aldrich). After incubating for 1 h at room temperature, the plates were washed 5 times with T-PBS. Next, 100 µL of TMB liquid substrate (Sigma-Aldrich) was added to each well. After color development for 30 min at room temperature, the reaction was stopped with 2 N H_2_SO_4_ and the absorbance at 405 nm (OD_405_) was measured.

### Measurement of serum vibriocidal responses

Serum vibriocidal antibody titers against *V. cholerae* O1 were measured by in vitro microdilution assay, as previously described [13]. Briefly, the complement activity of the mouse sera was inactivated by heating sera to 56 °C for 1 h. Next, 50 μL of diluted heat-inactivated sera in PBS was added to 96-well tissue culture plates containing 10^8^ CFU/mL *V. cholerae* O1 in sterile PBS and 22% guinea pig complement (Sigma-Aldrich). Then, 150 μL of BHI broth was added to each well and incubated for 2 h at 37 °C. The absorbance at OD_600_ was measured. The vibriocidal titer was assessed as the dilution of serum causing a 50% reduction in OD compared with that of control wells without serum.

### Cytokine assays

Serum TNF-α, IL-4, IL-5, IL-6, IL-10, TGF-β, and BAFF were measured using the ELISA assay (R&D Systems, USA). Briefly, 96-well microtiter plates (Nunc, USA) were coated with the cytokine-specific capture antibody and incubated overnight at 4 °C, washed with 0.5% Tween-PBS (T-PBS), and blocked with PBS + 3% bovine serum albumin (Sigma-Aldrich). Then sample from five mice in each group was incubated for 2 h at RT, washed 3X with T-PBS, and biotinylated cytokine-specific detection antibody was incubated (1 h, RT). Plates were washed and incubated with streptavidin for 2 h at RT. Plates were then washed five times with T-PBS, and TMB substrate was added (100 µL/well; 30 min. at RT). Color development was stopped with 2 N H_2_SO_4_, and the absorbance at 405 nm (OD 405) was measured using BioTek™ ELx800™ absorbance microplate reader.

### Investigation of CD4^+^ T-cell population

Two weeks after the last immunization, mouse spleens were removed, homogenized, and cells were suspended in 3 mL of PBS containing 2% FBS, as previously described [36]. Red blood cells (RBC) were lysed using the RBC lysis buffer (eBioscience, USA), washed, centrifuged, and re-suspended in PBS. The CD4^+^ T-cell population was accessed by flow cytometry using a CD4-specific monoclonal antibody (Santa Cruz, USA).

### Bacterial challenge

Infection challenge experiments were performed using 3- to 5-day-old neonatal BALB/c mice, each group comprising 5 mice. Briefly, neonatal mice were orally infected with 25 µL *V. cholerae* O1 (10^8^ CFU/mL) plus 25 µL immunized and nonimmunized mouse sera. In survival rate studies, all immunized and challenged mice were monitored every 6 h for 48-h post-challenge.

### Statistical analysis

All statistical analyses were performed using GraphPad Prism 6 (GraphPad Software, Inc., USA). Differences between groups in MTT results and cytokine responses were determined by two-way analysis of variance (two-way ANOVA) followed by Tukey’s post hoc test for multiple comparisons. The antibody titers were scored as endpoint titers for each sample for all examined groups. Samples that did not produce a signal in the ELISA at the starting dilutions were considered negative but considered as 1 for the endpoint titer; thus, log_10_ transformation could be performed and was analyzed by one-way analysis of variance with Tukey’s multiple comparison test.

Survival data for different mouse groups were analyzed using the Mantel–Cox log-rank test. All results are expressed as the mean ± standard deviation (SD). A *P* value < 0.05 was considered statistically significant.

## Results

### Characterization of MSCs

**Fig. 1:**
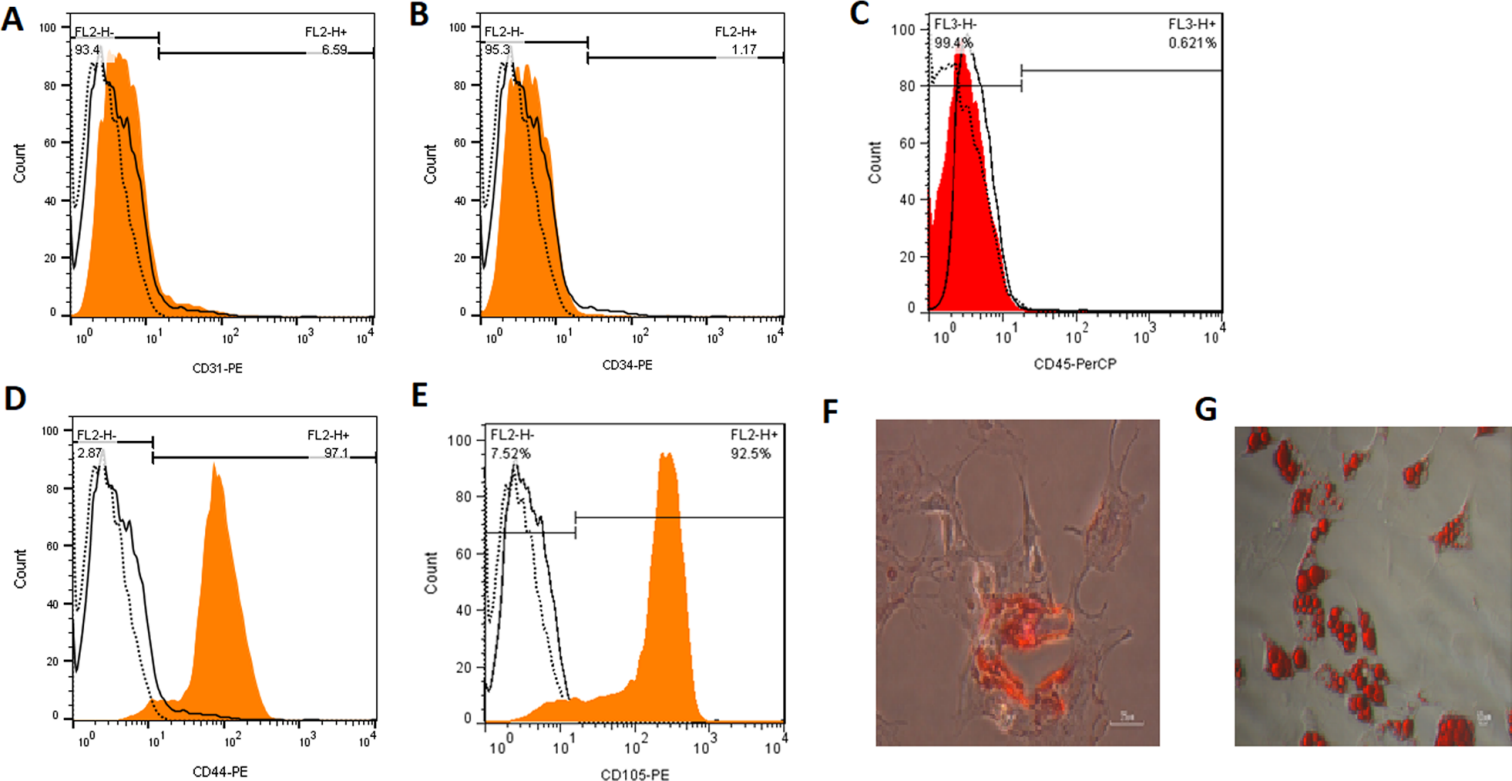
Flow cytometric analysis of the cell-surface markers on mesenchymal stem cells (MSCs). The *X*-axis shows the mean fluorescence intensity, and the *Y*-axis shows the cell number. MSCs were negative for CD31 (**A**), CD34 (**B**), and CD45 (**C**) and positive for MSC-specific cell-surface markers, including CD44 (**D**) and CD105 (**E**). Differentiation potential of MSCs into osteocytes (**F**) and adipocytes (**G**). Color-shaded area on the histogram is the MSC group

MSCs were characterized using specific monoclonal antibodies to MSC-specific surface antigens and by their in vitro adipogenic and osteogenic differentiation. MSCs were negative for CD31, CD34, and CD45 as standard hematopoietic surface markers (Fig. 1A–C) but positive for CD44 and CD105 (Fig. 1D, E). Oil red O and alizarin red S staining demonstrated the differentiation potential of MSCs into osteocytes and adipocytes (Fig. 1F, G).

### Determination of the optimal dose and timing of LPS preconditioning of MSCs

**Fig. 2:**
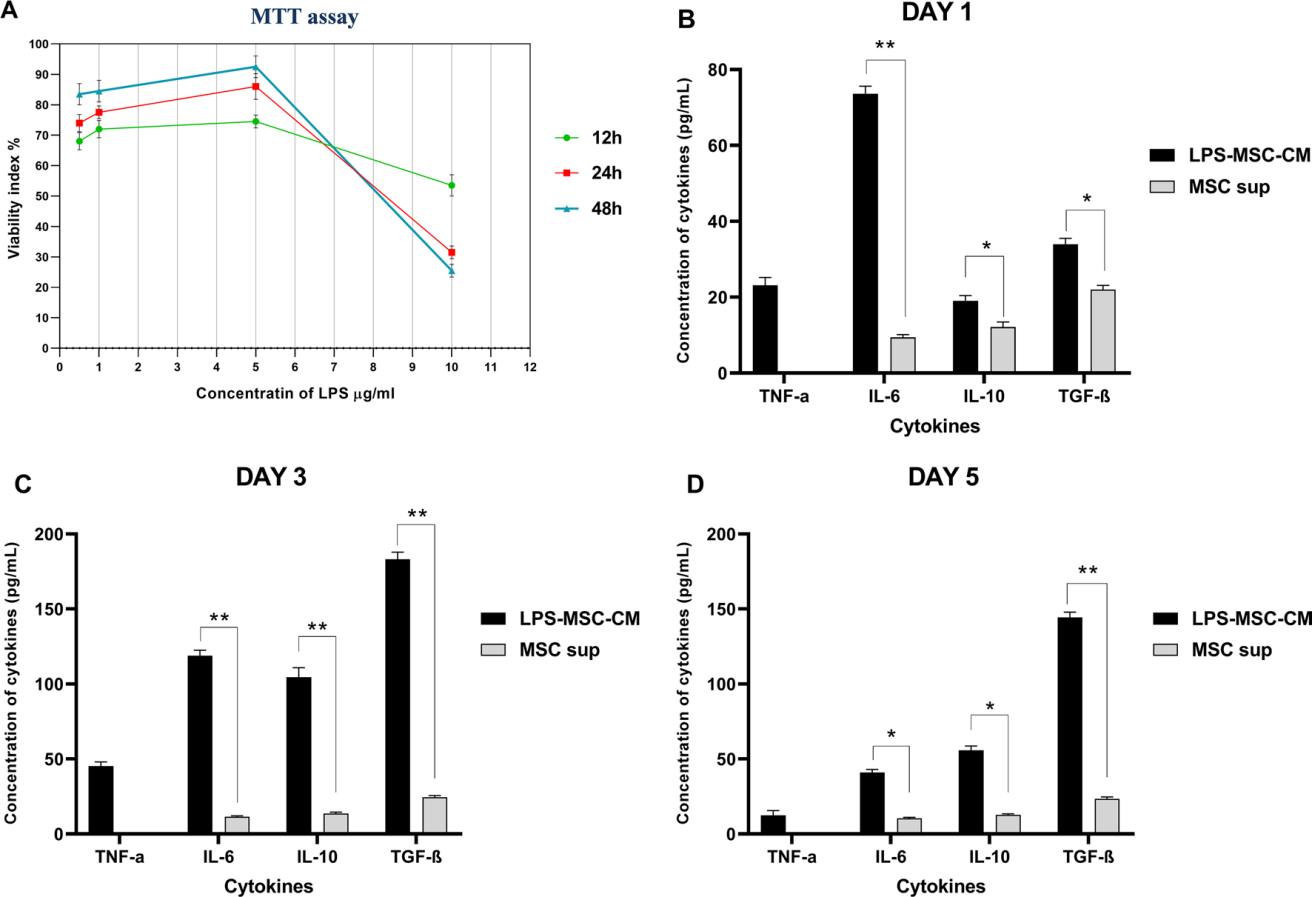
Determination of the optimal dose and timing of LPS preconditioning of MSCs. The cytotoxic effects of different doses of LPS on mesenchymal stem cell (MSC) viability (**A**). The concentration of IL-10, TGF-β, TNF-α, and IL-6 levels in the supernatant of LPS-conditioned MSCs on 1 (**B**), 3 (**C**), and 5 (**D**) days. Data are presented as mean ± SD of three independent experiments

The MTT assay was used to determine the most effective cytoprotective dose of LPS for preconditioning MSCs. As is shown in Fig. 2A, treatment of MSCs with 5 µg/mL LPS for 48 h significantly improved cell viability and proliferation compared to treatment times of 12 and 24 h (*P* < 0.05). In addition, preconditioning MSCs with 5 µg/mL LPS significantly increased the cell viability compared to other groups (*P* < 0.05). MSC viability in the presence of 1 µg/mL of LPS was significantly higher than that in the presence of 10 µg/mL LPS among after 12, 24, and 48 h (*P* < 0.05; Fig. 2A). These results indicate that the most effective cytoprotective dose of LPS for preconditioning MSCs was 5 µg/mL. To determine the optimal time for LPS preconditioning, MSCs were treated with 5 µg/mL of LPS as a cytoprotective dose and the concentration of pro- and anti-inflammatory cytokines were investigated for 1, 3, and 5 days. Following the preconditioning, the anti-inflammatory cytokine concentration increased, causing the elevated inflammatory cytokine concentration to decrease over time. As is shown in Fig. 2C, D, at day 3, the inflammatory cytokines levels of TNF-α and IL-6 in the LPS-MSC‐CM reached a peak compared with those at days 1 and 5 (*P* < 0.05). Moreover, the anti-inflammatory cytokines levels of IL-10 and TGF-β reached a peak at day 3 (*P* < 0.05; Fig. 2B). Importantly, the amount of anti-inflammatory cytokines decreased with reducing levels of inflammatory cytokines in the LPS-MSC‐CM over time (Fig. 2C, D). Also, there were significant differences in IL-6, TNF-α, IL-10, and TGF-β levels between the LPS-MSC-CM and MSC sup (*P* < 0.05; Fig. 2C, D).

### LPS + LPS‐MSC‐CM vaccine induces high IgG and IgA levels

**Fig. 3:**
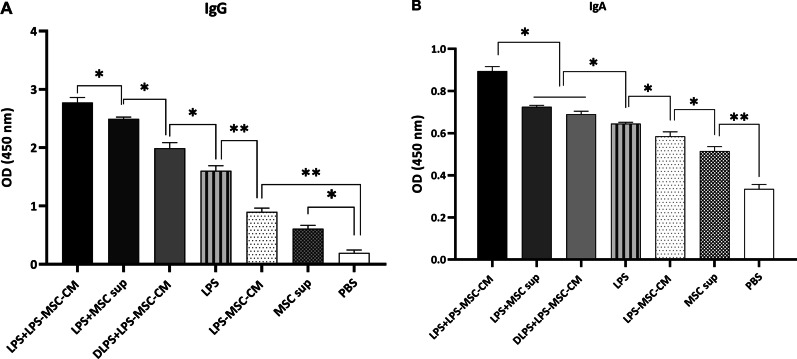
Effects of LPS‐MSC‐CM immunization on IgG (**A**) and IgA (**B**) levels against *Vibrio cholerae* LPS. Values are presented as mean ± SD based on five mice in each group. *P < 0.05 and **P < 0.01 indicate the groups that were significantly different

To evaluate humoral responses induced by the LPS + LPS‐MSC‐CM vaccine, IgG and IgA titers against the *V. cholerae* LPS were investigated among immunized mice. The antibody levels were significantly higher in the vaccine group than any other group (*P* < 0.05; Fig. 3A, B). There were significant differences in the IgG levels among the LPS + MSC sup, DLPS + LPS-MSC-CM, LPS, LPS-MSC‐CM, MSC sup, and PBS groups (*P* < 0.05; Fig. 3A). The IgA levels in the LPS + MSC sup or DLPS + LPS-MSC-CM-immunized mice saliva were similar (*P* > 0.05; Fig. 3B); however, these were significantly higher than those in the LPS, LPS-MSC‐CM, MSC sup, and PBS groups (*P* < 0.05; Fig. 3B). There were significant differences in the IgA levels among the LPS, LPS-MSC‐CM, MSC sup, and PBS groups (*P* < 0.05; Fig. 3B).

### LPS + LPS‐MSC‐CM-immunized mouse serum shows high vibriocidal activity against *V. cholerae*

**Fig. 4:**
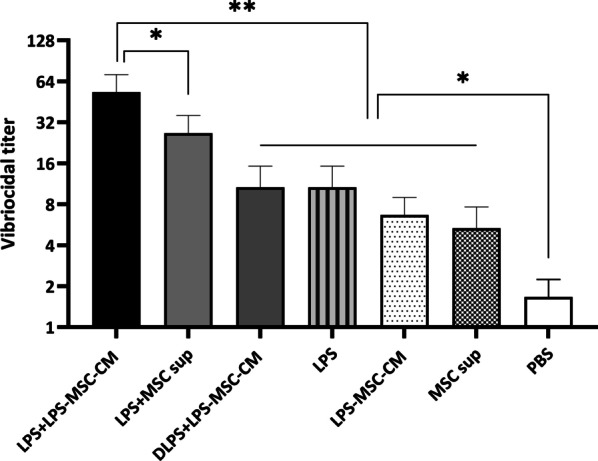
Analysis of vibriocidal activity of sera from immunized and nonimmunized mice. Values are presented as mean ± SD based on 5 mice in each group. *P < 0.05 and **P < 0.01 indicate the groups that were significantly different

As vibriocidal activity is crucial for protection against *V. cholerae* infection, the in vitro vibriocidal activity of sera from immunized and nonimmunized mice was evaluated. As is shown in Fig. 4, sera from LPS + LPS‐MSC‐CM-immunized mice demonstrated the highest vibriocidal activity against *V. cholerae,* with titer a 1:64 (*P* < 0.05). The vibriocidal activity of sera in the LPS + MSC sup group was significantly higher than that in the DLPS + LPS-MSC-CM, LPS, LPS-MSC‐CM, and MSC sup groups (*P* < 0.01; Fig. 4). There were no significant differences in the vibriocidal activity of sera among the DLPS + LPS-MSC-CM, LPS, LPS-MSC‐CM, and MSC sup groups; however, they were significantly higher than PBS group (*P* > 0.05; Fig. 4).

### LPS + LPS‐MSC‐CM immunization increase cytokines that regulate humoral immunity

**Fig. 5:**
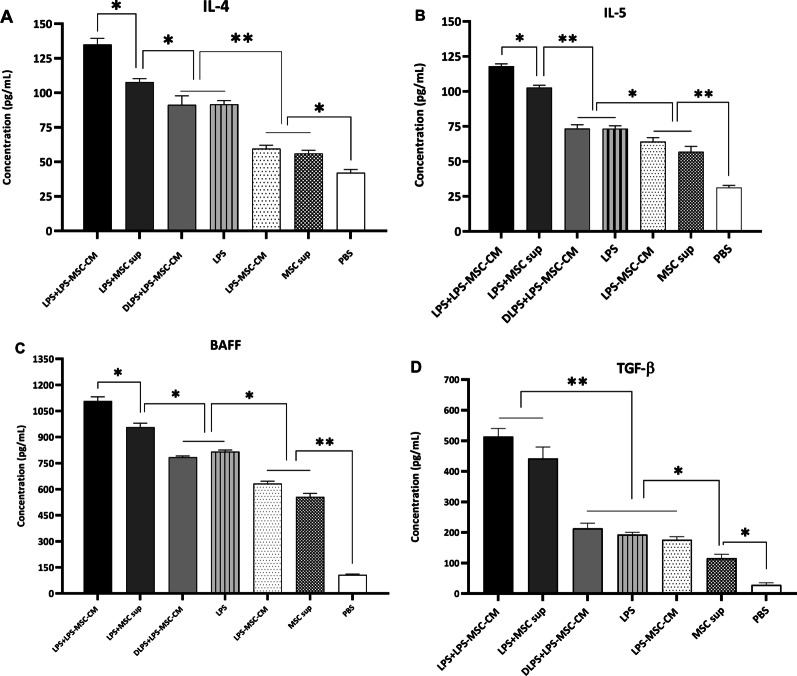
Comparison of cytokine production from immunized and nonimmunized mice. Two weeks after the final immunization, sera were obtained from immunized and nonimmunized mice, and the levels of IL-4 (**A**), IL-5 (**B**), BAFF (**C**), and TGF-β (**D**) were measured. Data are presented as mean ± SD based on 5 mice in each group. *P < 0.05 and **P < 0.01 indicate the groups that were significantly different

To study the effects of the LPS + LPS‐MSC‐CM vaccine on cytokines regulating humoral immunity, 2 weeks after the last immunization, serum IL-4, IL-5, BAFF, and TGF-β levels were evaluated among immunized and nonimmunized mice. As is shown in Fig. 5A–C, immunization with the LPS + LPS‐MSC‐CM significantly increased IL-4, IL-5, and BAFF levels in the vaccine group compared to other groups (*P* < 0.05). Serum IL-4, IL-5, and BAFF levels in the LPS + MSC sup group were significantly higher than the DLPS + LPS-MSC-CM, LPS, LPS-MSC‐CM, and MSC sup groups (*P* < 0.05; Fig. 5A–C). There were no significant differences in IL-4, IL-5, and BAFF levels between DLPS + LPS-MSC‐CM- and LPS -immunized mouse sera (*P* > 0.05), although these were significantly higher than the LPS-MSC‐CM, MSC sup, and PBS groups (*P* < 0.05; Fig. 5A–C). In addition, there were no significant differences in serum IL-4, IL-5, and BAFF levels between the LPS‐MSC‐CM- and MSC sup groups (*P* > 0.05), although these were significantly higher than the PBS group (*P* < 0.05; Fig. 5A–C). There were no significant differences in serum TGF-β level between the LPS + LPS‐MSC‐CM and LPS + MSC sup groups (*P* > 0.05), although these were significantly higher than other groups (*P* < 0.01; Fig. 5D). The serum TGF-β level among the DLPS + LPS‐MSC‐CM, LPS, and LPS‐MSC‐CM groups was significantly higher than MSC sup and PBS groups (*P* < 0.05; Fig. 5D). Moreover, serum TGF-β level in the MSC sup-immunized group was significantly higher than the PBS group (*P* < 0.05; Fig. 5D).

### LPS + LPS‐MSC‐CM immunization increases CD4^+^ T cells

**Fig. 6:**
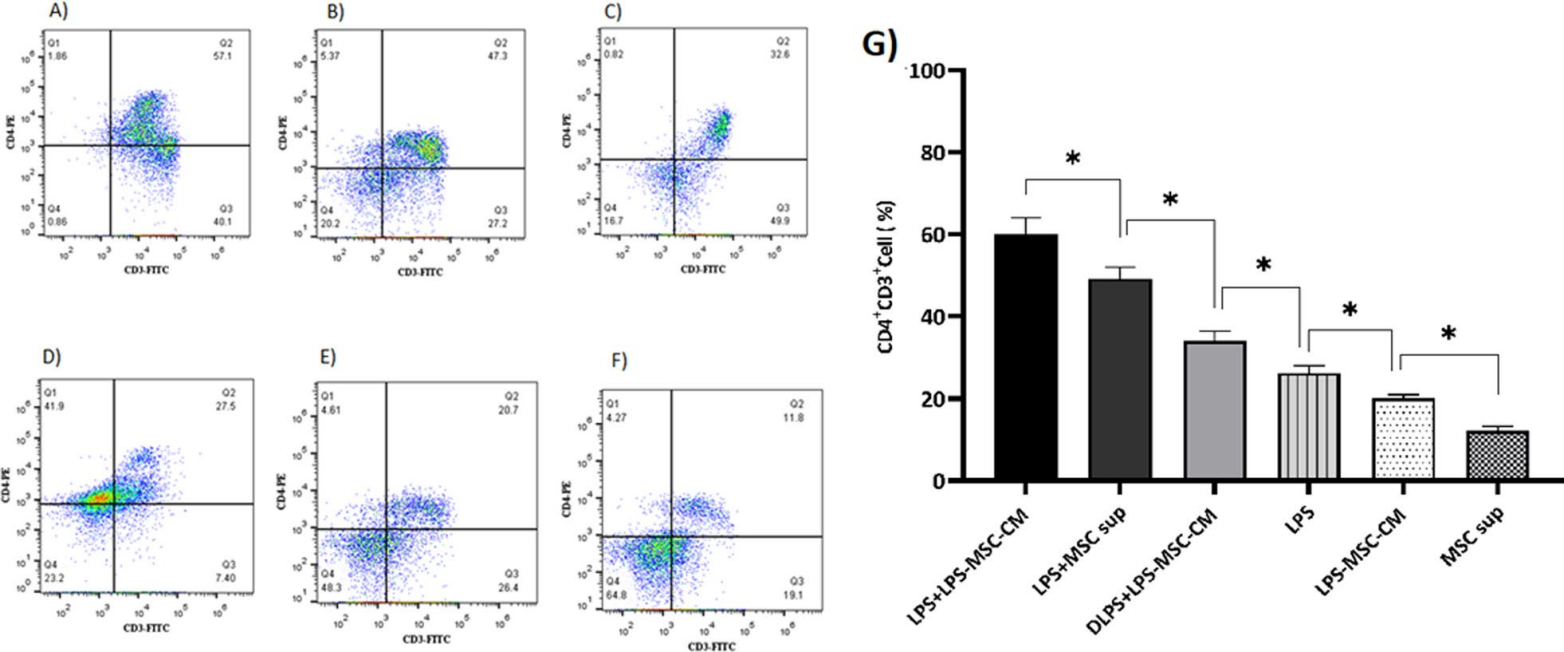
Effects of LPS‐MSC‐CM immunization on the CD4^+^ T-cell population. Flow cytometric analysis of CD4^+^ T cells from LPS + LPS-MSC-CM (**A**), LPS + MSC sup (**B**), DLPS + LPS-MSC-CM (**C**), LPS (**D**), LPS-MSC-CM (**E**), and MSC sup (**F**) immunized mice. The percentage of CD4^+^ T cells collected from immunized and nonimmunized mice (**G**). Values are presented as mean ± SD based on 5 mice in each group. *P < 0.05 indicates the groups that were significantly different

We determined whether the LPS + LPS‐MSC‐CM vaccine was effective in increasing the CD4^+^ T-cell population by flow cytometry using a CD4-specific monoclonal antibody. As is shown in Fig. 6A–G, the CD4^+^ T-cell population was increased in the LPS + LPS‐MSC‐CM group compared to other groups (*P* < 0.05). There were significant differences in CD4^+^ T-cell numbers among the LPS + MSC sup, DLPS + LPS‐MSC-CM, LPS, LPS-MSC‐CM, and MSC sup groups (*P* < 0.05; Fig. 6A–G).

### LPS + LPS‐MSC‐CM immunization modulates inflammatory and anti-inflammatory responses

**Fig. 7:**
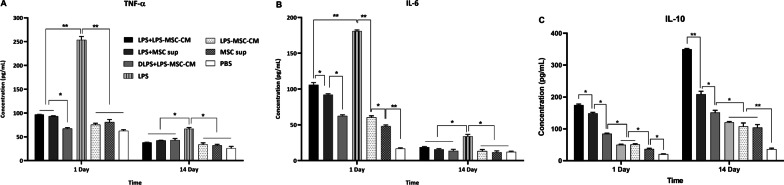
Serum TNF-α (**A**), IL-6 (**B**), IL-10 (**C**) levels of immunized and nonimmunized mice on the 24 h after each immunization. Values are presented as mean ± SD based on 5 mice in each group. *P < 0.01 and **P < 0.01 indicate the groups that were significantly different

To study the immunomodulatory effects of the LPS + LPS‐MSC‐CM vaccine on inflammatory and anti-inflammatory cytokines, 24 h after each immunization, we evaluated serum TNF-α, IL-6, and IL-10 levels in immunized and nonimmunized mice. TNF-α and IL-6 levels on days 1 and 14 were significantly higher in the LPS group than other groups (*P* < 0.05; Fig. 7A, B). The DLPS + LPS‐MSC‐CM-immunized group demonstrated the lowest TNF-α level on day 1 among immunized and nonimmunized mice (*P* < 0.05; Fig. 7A). There was no significant difference in the TNF-α level on days 1 and 14 among the DLPS + LPS‐MSC‐CM, LPS + MSC‐CM, MSC sup, and PBS groups (*P* > 0.05; Fig. 7A). As is shown in Fig. 7B, serum IL-6 was highest in the LPS group compared to other groups (*P* < 0.05). The serum IL-6 level on day 1 in the LPS + LPS-MSC‐CM group was significantly higher than the DLPS + LPS-MSC‐CM, LPS + MSC sup, LPS-MSC‐CM, MSC sup, and PBS groups (*P* < 0.05; Fig. 7B). There were no significant differences in serum IL-6 levels on day 1 between the DLPS + LPS-MSC‐CM and LPS-MSC‐CM groups (*P* > 0.05; Fig. 7B), although these were significantly higher than the MSC sup and PBS groups (*P* < 0.05; Fig. 7B). Moreover, serum IL-6 levels on day 1 in the MSC sup group were significantly higher than the PBS group (*P* < 0.05; Fig. 7B). As is shown in Fig. 7C, LPS + LPS‐MSC‐CM immunization significantly increased IL-10 levels compared to other treatments (*P* < 0.05). The serum IL-10 level on days 1 and 14 in the LPS + MSC sup group was significantly higher than in the DPS + LPS‐MSC‐CM, LPS, LPS‐MSC‐CM, MSC sup, and PBS groups (*P* < 0.05; Fig. 7C). The IL-10 level was significantly increased in the DLPS + LPS-MSC‐CM group compared to the LPS, LPS-MSC‐CM, MSC sup, and PBS groups (*P* < 0.05; Fig. 7C). The serum IL-10 level in the LPS, LPS-MSC‐CM, and MSC sup groups was similar (*P* > 0.05), although the level was significantly higher than the PBS group (*P* < 0.05; Fig. 7C).

### LPS + LPS‐MSC‐CM immune sera protect infant mice against V. cholerae infection

**Fig. 8:**
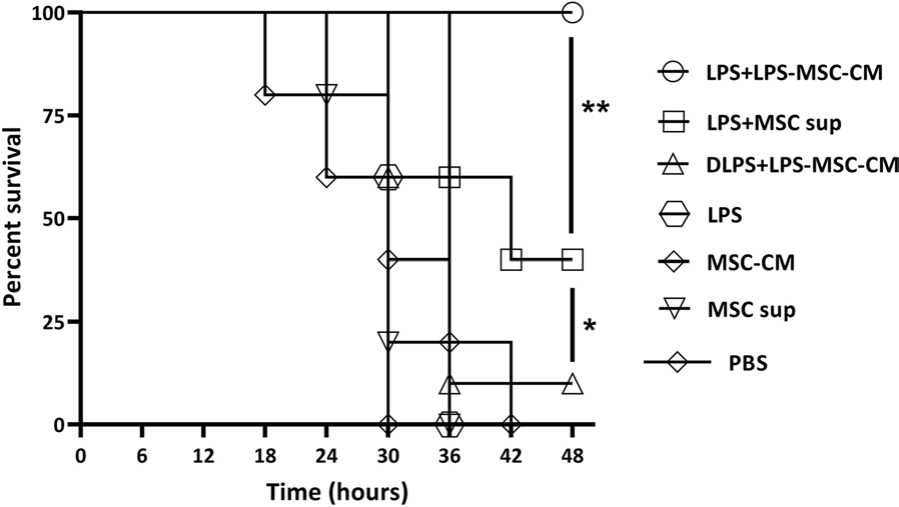
Survival rates of passively immunized neonatal mice (*n* = 5 mice/group) challenged with *Vibrio cholerae* O1. *P < 0.05 and **P < 0.01 by Mantel–Cox log-rank test

We evaluated the efficacy of serum from LPS + LPS‐MSC‐CM-immunized mice to protect infant mice against a lethal dose of *V. cholerae*. As is shown in Fig. 8, LPS‐MSC‐CM antisera completely protected neonatal mice from *V. cholerae* O1 challenge (100% survival; *P* < 0.01). Sera from the LPS + MSC sup group significantly protected mice against *V. cholerae* O1 (40% survival) compared to sera from the DLPS + MSC‐CM, LPS, LPS-MSC‐CM, MSC sup, and PBS groups (*P* < 0.05; Fig. 8). There were no significant differences in survival rates of neonatal mice that received sera from DLPS + MSC‐CM, LPS, LPS-MSC‐CM, or MSC sup groups (*P* > 0.05; Fig. 8).

## Discussion

The goal of this study was to evaluate the immunomodulatory effects of LPS-MSC-CM on *V. cholerae* LPS as a vaccine candidate in the murine model. LPS as a protective immunogen for cholera vaccine plays a key role in the pathogenesis of *V. cholerae* by initiating and maintaining the inflammatory response, which impairs the immune system because it reduces the protective anti-inflammatory responses and increases *V. cholerae* invasion and intestinal pathology [2, 15, 16, 37]. MSCs have been used to treat bacterial infection-induced sepsis by reducing severe inflammatory response without severe immunosuppression and promoting bacterial clearance [38, 39]. Recently, we reported the robust antibacterial and antibiofilm activity of the MSC supernatant against *V. cholerae* [40]. It appears that the antimicrobial and properties of MSCs were modulated through the TLR-4 signaling pathways by LPS [41]. In addition, other studies have demonstrated that preconditioning of MSCs with TLR-like receptor agonists can alter the cytokine profile [42]. For instance, treating MSCs with TLR3 ligands prolonged the neutrophil survival, which is essential for clearing a bacterial infection [43]. It has been documented that MSCs show two diverse phenotypes including pro-inflammatory MSC type 1 (MSC1) and immunosuppressive MSC type 2 (MSC2) phenotypes based on which TLRs are activated at different exposure time with TLR ligands. A previous study showed MSC1 and MSC2 phenotype are induced after TLR4 activation with LPS by 1 h and TLR3 activation with Poly(I:C) by 1 h, respectively [44]. In our study, MSCs at different exposure times with LPS display pro- or anti-inflammatory cytokine production, indicating that the dual phenotype, MSC1 or MSC2, may have adopted functions in the presence of immunomodulators in the microenvironment [45–47]. The molecular mechanism on how exactly TLR4 responds to the same pro-inflammatory stimuli at different exposure times remains to be investigated. It seems anti-inflammatory phenotype of MSCs can be induced by long exposure to LPS and MyD88-independent TLR4 signaling pathways [48, 49]. In Myd88-independent pathway, activation of TIR-​domain-containing adapter-inducing interferon-β (TRIF) and tumor necrosis factor (TNF) receptor-associated factor 3 (TRAF3), leads to the activation of IRF3, which induces the expression of anti-inflammatory cytokines [48]. In MyD88-dependent signaling pathway, activation of activator protein 1 (AP1) transcription factor followed by the induction of mitogen-activated protein kinase (MAPKs), Janus kinase-phosphoinositide 3-kinase (JAK-PI3K), and nuclear factor kB (NF-kB), lead to induction of pro-inflammatory cytokine production. It has recently been demonstrated that the treatment of MSCs with LPS for 1 h induces a more robust MSC1 phenotype with high levels of IL6 in comparison with LPS-treated MSCs for 48 h, which demonstrates a potent immune-suppressive MSC2 phenotype [49]. It seems the MSC2 phenotype induced by long exposure to LPS is fully or partially dependent on the TLR4 pathway [49]. It seems that the stimulation of MSCs with LPS at different exposure times is associated with MSCs phenotype conversion, which highlights the immunomodulatory potential of MSCs for treatment of infectious and inflammatory diseases. In the present study, we demonstrated that the LPS-MSC-CM regulates the balance of the production of inflammatory and anti-inflammatory mediators in an appropriate time and amount, which plays an important role in protecting the host against *V. cholerae* infection. In the present study, we showed that the LPS + LPS-MSC-CM vaccine protects the murine model against *V. cholerae* infection. Our data revealed that LPS + LPS-MSC-CM-immunized immune sera protected neonatal mice against the lethal challenge of *V. cholerae* and that their survival rates (100%) were remarkably increased compared to other study groups, indicating the LPS + LPS-MSC-CM efficiently surpasses LPS or DLPS series of *V. cholerae* vaccines. Active immunization with the LPS + LPS-MSC-CM effectively stimulated a strong humoral response, which was associated with elevated levels of IL-4, IL-5, IL-6, BAFF, and TGF-β cytokines and IgG and secretory IgA antibodies. Cholera infection or vaccination increased the total serum IgG and secretory IgA levels, which play an important role in providing protection against *V. cholerae* infection [2, 10–12]. Importantly, IgG and IgA antibody titers elevated considerably in the LPS + LPS-MSC-CM group compared to the LPS-alone group, suggesting the immune-modulating effects of the LPS-MSC-CM, which increases the efficacy of the LPS vaccine. Sera from LPS + LPS‐MSC‐CM-immunized mice demonstrated the highest IgG level, which has critical role in vibriocidal activity [50]. Moreover, the highest vibriocidal activity was observed in sera from LPS + LPS‐MSC‐CM-immunized mice and their vibriocidal titer (1:64), demonstrating the efficacy of LPS + LPS-MSC-CM in inducing vibriocidal antibodies to LPS or DLPS series of *V. cholerae* vaccines. However, the vibriocidal titer has been consistently related to protection against *V. cholerae* regardless of age. Children demonstrate robust vibriocidal responses to cholera vaccines but experience lower vaccine efficacy and a shorter duration of protection compared to adults [51]. Moreover, baseline antibody titers among household contacts of patients infected with *V. cholerae* did not relate to the inhibition of colonization or any subsequent diseases [52]. In previously *V. cholerae*-infected volunteers with very low serum vibriocidal titer, a full clinical protection was demonstrated 3 years after challenge with wild-type *V.cholerae* [53]. Vibriocidal antibody titer declined in vaccinated children with inactivated whole-cell *V. cholera* after one-year post-vaccination*,* whereas protective efficacy was maintained for at least five years verifying a previous suggestion that serum vibriocidal antibody titer is not associated with protection [54–56]. It seems that vibriocidal antibody may not be an appropriate indicator for protective immunity against infection with *V. cholerae* 0139 serogroup [52]. It has also been demonstrated that the duration and extent of protection mediated by vibriocidal antibody and CTB-specific IgA decline more rapidly than the protection observed after natural infection, suggesting that other longer-lasting immunologic responses are necessary for protection [57]. In addition, IgA levels differ from the serum vibriocidal antibodies and are believed to mediate the protection also decreased approximately one-year luminal after vaccination [58]. Vibriocidal antibody response may be an alternative marker for specific secretory IgA responses directed against *V. cholerae* antigens on mucosal surfaces which are known as the primary mediators of protective immunity [52]. In patients with cholera, LPS-specific memory B cell responses are not dependent on T-cell recognition and activation and diminished more rapidly than compared to responses generated by T-dependent protein antigens including CTB and TcpA [59–61]. Cytokine secretion and co-stimulation of CD4^+^ T cells are associated with the duration and quality of memory B-cell responses to protein antigens [62, 63]. Also, in secondary lymphoid tissue, direct interaction of T and B cells helps CD40/CD40 ligand (CD40L) interaction, which have a critical role in B-cell proliferation and isotype switching as well as memory B-cell activation [62–64]. In accordance with previous studies, our result shows that active immunization with LPS + LPS-MSC-CM increased CD4^+^ T cells, which may have important role in long-lived memory B cells that respond to differentiation and proliferation upon re-exposure to *V. cholerae* antigens [65]. It has been recently demonstrated that memory T-cell responses to *V. cholerae* infection induce an anamnestic development and stability of memory B-cell responses mounted in the intestinal mucosa [61]. These findings are consistent with previous studies that reported CD4^+^ T cells are critical for long-term protection mediated by memory B cells and plasma cells that facilitate a rapid anamnestic response upon re-exposure inhibiting the infection before it causes illness [66]. Our results suggest that the vibriocidal titer and the ability of *LPS-*specific memory B cells to respond to re-exposure have important roles in protecting *V. cholerae*. In addition to lacking the immunologic priming of repeated exposures, this relationship between vibriocidal titers and protection may be influenced by other host factors that differentiate adults and children, including immunologic immaturity, higher levels of enteric enteropathy and malnutrition, differences in intestinal parasitic burdens, or differences in the gut microbiome, a newly recognized host factor that is important for the development of mucosal immune responses [67]. It has also been demonstrated that the enhanced production of IL-4 and IL-5 cytokines can contribute to the combat against *V. cholerae* infection by inducing vibriocidal antibodies via regulating antibody isotype switching and promoting Th2-cell differentiation [50, 68–70]. Recent studies have also reported that IL-4 and IL-5 cytokines induce the expression of serum IgA and secretory IgA, respectively, which activate the classical complement pathway to cause bacterial lysis and prevent the attachment of *V. cholerae* to intestinal epithelial cells [2, 71, 72]. It has been demonstrated that the initial CD4^+^ T-cell responses to *V. cholerae* infection or antigens have an important role in the induction of long-term protection against cholera through the development of long-lived memory B cells [59, 73]. Th1 or Th17 responses have been shown to play an important role in providing protective immunity against infections by invasive mucosal pathogens [74–77]. In the murine model, we show that active immunization with LPS + LPS-MSC-CM can induce the production of BAFF, which mediates activation and maturation of splenic B cells, and ultimately their differentiation into plasma cells to produce immunoglobulins that are crucial for the host immunity to *V. cholerae* [2, 71, 72, 78–81]. This outcome is consistent with reports that suggest BAFF enhances the differentiation of memory B cells and IgA-producing cells, which could inhibit the binding of *V. cholerae* to host epithelial cells by enhancing the specific IgA response against LPS [71, 72, 81]. We also found that immunization with the LPS + LPS-MSC-CM induced IL-10 and TGF-β, which regulate proliferation and differentiation of B and T lymphocytes, as well as the production of vibriocidal antibodies [81–83]. Instead, infection with *V. cholerae* primed Th1 and Th17 responses, with a shift toward Th1 to Th2 CD4^+^ T-cell responses [81–83]. IL-6, which acts as a critical bridge between innate and adaptive immune systems as well as promoting B-cell IgA class switching and Th17 differentiation (Th17 lineage), has been associated with the development of long-term immunologic memory and responses [73]. An appropriate level of IL-6 plays a critical role in activating and differentiating B lymphocytes into plasma cells to produce IgG2a and mucosal IgA antibodies, which may be associated with decreased host susceptibility to *V. cholerae* infection [84]. It has also been revealed that due to the presence of CtxB in the whole cell vaccine formulation, the most CD4^+^ T cells responses skewed to regulatory T cells (Tregs) compared to the TH1 and TH17 related to short-term immunity to cholera [85]. CD4^+^ T-cell responses to *V. cholerae* following the WC-CTB immunization reduced or skewed toward development of a Th2 T-cell phenotype [85]. Also, CtxB promotes differentiation of IL-10-producing Tregs and inhibits Th1, Th2, and Th17 responses, which resulted in tolerance to vaccine antigens [86–88]. Moreover, this may seem to explain why the whole cell vaccine without CtxB has provided long-term immune protection [89–91]. In this study, we observed LPS + LPS‐MSC‐CM immunization increases CD4^+^ T cells, which may result in long-lasting immune responses against *V. cholerae*. In light of these findings, a thorough assessment of CD4^+^ T cells and B cell population involved in maintaining long-term immunity to cholera after “LPS + LPS‐MSC‐CM” vaccine under controlled clinical settings seems warranted. Thus, increased levels of secretory IgA following increased IL-4, IL-5, and BAF levels in LPS + LPS-MSC-CM-immunized mice have a critical function in reducing *V. cholerae* colonization by inhibiting adhesion of *V. cholerae* and increasing complement-mediated bacterial lysis. Importantly, anti-LPS antibodies can also agglutinate bacteria in the mucosal area and reduce the possibility of interaction with the intestinal epithelium [70, 92]. We also found that immunization with the LPS + LPS-MSC-CM induced IL-10 and TGF-β, which regulate proliferation and differentiation of B and T lymphocytes, as well as the production of vibriocidal antibodies [81–83]. An appropriate level of IL-6 plays a critical role in activating and differentiating B lymphocytes into plasma cells to produce IgG2a and mucosal IgA antibodies, which may be associated with decreased host susceptibility to *V. cholerae* infection [84]. In the murine model, we show that active immunization with LPS + LPS-MSC-CM can induce the production of BAFF, which mediates activation and maturation of splenic B cells, and ultimately their differentiation into plasma cells to produce immunoglobulins that are crucial for the host immunity to *V. cholerae* [2, 71, 72, 78–81]. This outcome is consistent with reports that suggest BAFF enhances the differentiation of memory B cells and IgA-producing cells, which could inhibit the binding of *V. cholerae* to host epithelial cells by enhancing the specific IgA response against LPS [71, 72, 81]. Thus, increased levels of secretory IgA following increased IL-4, IL-5, and BAF levels in LPS + LPS-MSC-CM-immunized mice have a critical function in reducing *V. cholerae* colonization by inhibiting adhesion of *V. cholerae* and increasing complement-mediated bacterial lysis. Importantly, anti-LPS antibodies can also agglutinate bacteria in the mucosal area and reduce the possibility of interaction with the intestinal epithelium [70, 92].

LPS induced overexpression of the inflammatory cytokines IL-6 and TNF-α and inhibited IL-10 and TGF-β, which is associated with pathological effects and tissue damage following *V. cholerae* infection [2, 15, 16]. Analysis of serum cytokines of immunized mice revealed that the LPS + LPS-MSC-CM vaccine modulates the systemic IL-6 and TNF-α levels, which are an indicator of the systemic inflammatory response status [93]. We also found that vaccination with LPS + LPS-MSC-CM hinders the LPS-induced harmful decrease in IL-10 level by modulating the systemic IL-6 and TNF-α levels, along with increasing IL-10 levels, which inhibit adverse inflammatory responses following the *V. cholerae* infection. In accordance with previous studies, LPS-MSC-CM has potential to reduce LPS-induced overexpression of inflammatory cytokines by increasing the anti-inflammatory cytokine IL-10, which may decrease *V. cholerae* intestinal pathology and invasion by preventing uncontrolled inflammatory response to infectious stimuli [2, 15, 94]. Consistent with our data, intraperitoneal administration of MSCs had immunomodulatory effects on the inflammatory response by increasing IL-10 in septic mice [39]. More recent evidence suggests that MSCs, as an attractive therapeutic candidate, prevent apoptosis of epithelial cells and beneficially modulate the inflammatory cytokines TNF-α and IL-6 and the anti-inflammatory cytokines TGF-β and IL-10 [24, 28, 38]. Conversely, IL-10 overexpression may induce a temporary immune system suppression, which increases the susceptibility of the host to bacterial infections [83]. In the present study, we demonstrated that the LPS-MSC-CM regulates the balance of production inflammatory and anti-inflammatory mediators in an appropriate time and amount, which plays an important role in protecting the host against *V. cholerae* infection.

## Conclusions

We demonstrated that a new LPS + LPS-MSC-CM vaccine can be appropriate as a therapeutic option against *V. cholerae* infections. The vaccine modulates inflammatory and anti-inflammatory responses and elicits robust protective humoral immune responses by increasing vibriocidal antibodies that protect neonate mice from *V. cholerae* infection. In light of these findings, an exhaustive evaluation of the LPS + LPS-MSC-CM vaccine against broad *V. cholerae* clinical isolates is warranted.

## Supplementary Information

**Additional file 1**. Silver-stained SDS-PAGE of extracted LPS.

## Data Availability

The datasets used and/or analyzed during the current study are available from the corresponding author on reasonable request.

## Abbreviations

LPS: Lipopolysaccharide
MSCs: Mesenchymal stem cells
CM: Conditioned medium
BAFF: B-cell activating factor
WHO: World Health Organization
MDR: Multi-drug resistance
BHI: Brain heart infusion
LB: Luria–Bertani
CFU: Colony-forming units
DLPS: Detoxified lipopolysaccharide
MTT: 3-(4,5-Dimethylthiazol-2-yl)-2,5-diphenyltetrazolium bromide
DMSO: Dimethyl sulfoxide
