# Supplementary figures and images for: Immunomodulatory effects of mesenchymal stem cell-conditioned media on lipopolysaccharide of Vibrio cholerae as a vaccine candidate
Source: Stem Cell Res Ther. 2021 Nov 3;12:564. doi: 10.1186/s13287-021-02622-0 (PMC8567566; doi:10.1186/s13287-021-02622-0)

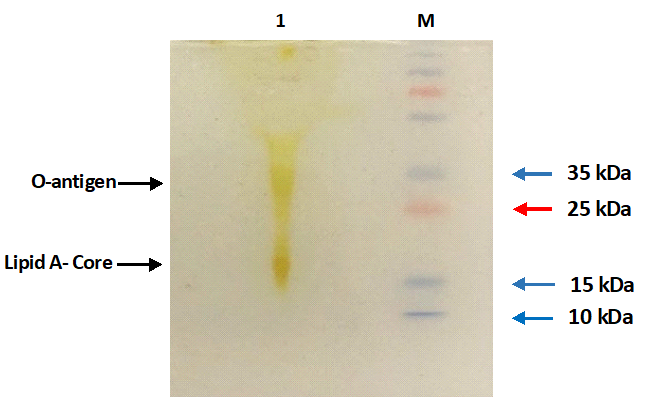

Supplement: Supplementary file 1 — Additional file 1. Silver-stained SDS-PAGE of extracted LPS. [file 13287_2021_2622_MOESM1_ESM.tif]
